# Mechanically Adjustable 4-Channel RF Transceiver Coil Array for Rat Brain Imaging in a Whole-Body 7 T MR Scanner

**DOI:** 10.3390/s24165377

**Published:** 2024-08-20

**Authors:** Sigrun Roat, Lena Nohava, Elmar Laistler

**Affiliations:** High Field MR Center, Center for Medical Physics and Biomedical Engineering, Medical University of Vienna, 1090 Vienna, Austrialena.nohava@meduniwien.ac.at (L.N.)

**Keywords:** magnetic resonance imaging, radiofrequency coil, rat imaging, ultra-high field, adjustable, transceiver, preclinical

## Abstract

Investigations of human brain disorders are frequently conducted in rodent models using magnetic resonance imaging. Due to the small specimen size and the increase in signal-to-noise ratio with the static magnetic field strength, dedicated small-bore animal scanners can be used to acquire high-resolution data. Ultra-high-field (≥7 T) whole-body human scanners are increasingly available, and they can also be used for animal investigations. Dedicated sensors, in this case, radiofrequency coils, are required to achieve sufficient sensitivity for the high spatial resolution needed for imaging small anatomical structures. In this work, a four-channel transceiver coil array for rat brain imaging at 7 T is presented, which can be adjusted for use on a wide range of differently sized rats, from infants to large adults. Three suitable array designs (with two to four elements covering the whole rat brain) were compared using full-wave 3D electromagnetic simulation. An optimized static B_1_^+^ shim was derived to maximize B_1_^+^ in the rat brain for both small and big rats. The design, together with a 3D-printed adjustable coil housing, was tested and validated in ex vivo rat bench and MRI measurements.

## 1. Introduction

Rat brain models are frequently used in preclinical studies of neurological disorders in humans [1,2]. Magnetic resonance imaging (MRI) and spectroscopy (MRS) are non-invasive, well-established research tools used to investigate anatomical and functional parameters in vivo. As a generally low-sensitivity imaging technique, improving its detection sensitivity is a central research question in MR. The size of the rat brain and the anatomical structures to be resolved set high requirements on the sensitivity of the measurement since the signal-to-noise ratio (SNR) is directly proportional to the voxel volume.

In addition to higher B_0_, which increases the overall spin polarization and therefore SNR, sensors for the MRI signal which are highly optimized for the specific application are required in order to achieve the necessary image quality. Thermal noise, as a limiting factor regarding the sensitivity of the experiment, appears as random intensity fluctuations resulting in image noise in MRI. The sample as well as circuit components, e.g., lossy conductors and capacitors inside radiofrequency (RF) coils and characteristics of other components in the MR signal receive chain, e.g., the preamplifier’s noise figure, contribute to the overall detected noise. For optimal performance, the total noise should be dominated by the noise originating from the sample and not the data acquisition process. RF coils, as the typical sensors for MRI and MRS, have the fundamental property that, as the size of the coil elements decreases, sensitivity increases. On the other hand, sample noise is reduced, which means that other noise sources become more prominent and a trade-off has to be found through optimization. One way to achieve this is to reduce coil noise by cooling the RF coils [3] or using superconducting coils [4], which reduces electrical resistance and can increase the SNR up to a factor of four [5]. However, such setups are complex, require lengthy scanner setup times, and require costly additional hardware. Further, its compatibility with in vivo preclinical imaging scenarios is limited, e.g., to maintain the body temperature of small animals located close to the cooled coil. Another option is to optimize the resonator size such that sample noise is still dominant, while the size-dependent penetration depth remains satisfactory. In combination with low-loss materials, this is a widely used solution for dedicated rat coils. Among those, volume coils [6,7,8,9,10] are known to achieve homogeneous transmit (B_1_^+^) and receive (B_1_^−^) fields but lack the high SNR needed in rat brain MRI. Single-channel surface coils generally provide higher signals [11,12,13], but depending on their size and targeted use case, might not cover the desired field of view (FOV). Implanted surface coils [14] alleviate the possible depth limitation and are known to increase the SNR, but involve a highly invasive procedure. A combination of volume coil transmitter and surface coil receiver was also found to be a suitable coil design for the superficially located rat brain [15]. Additionally, phased array coils [16] enable accelerated acquisition using parallel imaging techniques [17,18,19]. The use of dedicated rigid coil arrays is already common for rat brain [20], spine [21], knee [22] or whole-body MR imaging [23,24].

Another consideration in small RF coil designs for rodents is the influence of changing loading conditions, resulting in a frequency shift and therefore a potential signal loss due to imperfect impedance matching. Due to the high quality (Q-) factors of the small coil elements, the resonance has a narrow bandwidth, and even small frequency shifts result in considerably decreased power transfer due to insufficient matching. Additionally, the critical inter-element coupling is easily exceeded with high-Q coil elements, resulting in peak splitting. This behavior is even more severe at ultra-high field [25], since the Q-factor scales with the operating frequency. A possibility to account for changing loading conditions is the retuning and rematching of the RF coil for each setup [25], or ensuring that the sample, however different, poses a similar loading condition each time by form-fitting [26] or using flexible parts [27] or coils [28,29].

In this study, we present a rat brain RF coil at ultra-high field for use in a human whole-body 7 T system. The particularity of the coil design consists in a mechanically adjustable custom 3D-printed housing that allows the coil array to be closely fitted to the rat head, thereby accounting for different loading conditions. The coil should provide a compact and stable solution for the positioning of the rat in a human-sized scanner bore and an application-optimized transceiver array for rat brain MRI, circumventing the need for a dedicated preclinical (small animal) MR scanner.

## 2. Materials and Methods

### 2.1. Electromagnetic Simulation

All 3D electromagnetic simulations (EMSs) in this study were conducted using the finite difference time domain method in XFdtd 7.8 (Remcom, State College, PA, USA). Absorbing boundary conditions were implemented as 7 perfectly matched layers (PMLs). The cell size was forced in a grid region containing the RF coil and was set to 0.25 mm in each direction in order to fully resolve the coil structures and ensure conductor separation, especially in overlap regions. The total number of cells varied between ≈17.5 and ≈20 million for the 2-element and 4-element simulations. The full-wave 3D EMS yielded S-parameters and H- and E-field data. All lumped components were replaced with 50 Ω voltage sources in the 3D domain, in order to speed up the time-intensive, full-wave simulation [30]. The RF co-simulation step, in which all lumped components are modeled as linear networks, was implemented in ADS (Keysight Technologies, Santa Rosa, CA, USA). Capacitor, inductor, solder joint, and copper losses were incorporated as series resistances, with assigned values according to literature [31]. Data from 3D simulation and co-simulation were combined in Matlab 2017 (Mathworks). An in-house developed specific absorption rate (SAR) averaging algorithm, employing the quadratic form power correlation matrix formalism [32,33] and an ultra-fast convolution-based averaging algorithm [34], is used to calculate the safety-relevant 1 g average SAR.

Three suitable phased array coil designs for the target application of rat brain MRI that differed in the number of elements (2, 3 or 4) and arrangement were compared. The mean B_1_^+^ was evaluated in an ellipsoid region of interest (ROI) resembling the rat brain in terms of its dimensions and location from the coil array. Each coil design was simulated using a 1 mm thick wire modeled as a perfect conductor. Phase offsets for static B_1_^+^ shimming were derived using phase steps of 5°. To incorporate different loading conditions, the arrays were loaded by a large elliptic cylindrical phantom (semi-major axis a = 23 mm, semi-minor axis b = 17.5 mm) fitting the coil former, and a smaller cylindrical phantom (radius r = 16.5 mm) filled with tissue-simulating liquid (σ = 0.5 S/m, ε_r_ = 80). The ellipsoidal brain ROI’s volume was 4/3π × (23 × 12.5 × 25) mm^3^ = 30 mL and 4/3π × (17 × 9.3 × 18.5) mm^3^ = 12 mL for the two phantoms, respectively. Each design was tuned, matched, and decoupled separately. The performance was assessed via power and 1 g SAR efficiency, defined as $\bar{B_{1}^{+}}/\sqrt{P_{in}}$ and $\bar{B_{1}^{+}}/\sqrt{\max SAR_{1g}}$, respectively, in the aforementioned brain ROI. The best-performing array was chosen as the final design.

To evaluate the need for an adjustable housing, the performance of the final RF coil design was investigated with different loading conditions. The coil array’s tuning and matching capacitors were derived to yield minimal reflection (S_ii_) and transmission (S_ij_) coefficients while being loaded with an adult and infant rat. Since, in reality, coils are rarely retuned and rematched for each sample, this inherently results in non-optimal sensitivity with loading that differs from the case for which the coil is adjusted. This corresponds to the investigated configuration 1, assuming a non-adjustable housing for an adult rat and an infant rat with an air gap of 9 mm between the head and RF coil former as loading. In configuration 2, an adjustable housing that brings the coil array close to the rat’s head, regardless of its size, was assumed, which has the advantage of increasing sensitivity by making the coil load similar for all cases and reducing the distance between the coil and the sample.

The rat voxel model consisted of five different tissue types (skin, bone, subcutaneous fat, brain, and muscle), each assigned with frequency-dependent dielectric parameters [35]. For the final design, in addition to power and SAR efficiency, S-parameters were assessed.

### 2.2. Mechanically Adjustable Housing and RF Coil Design

**Figure 1:**
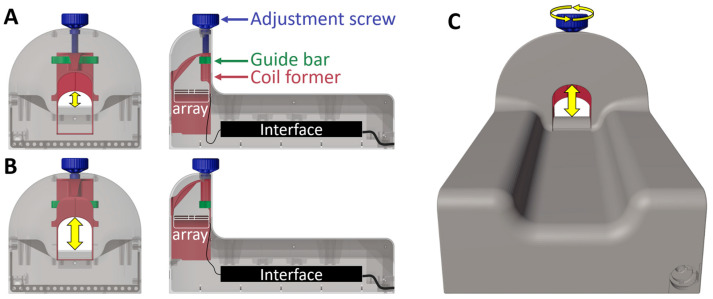
The 3D-printed housing for the smallest (**A**) and biggest (**B**) configuration possible. A 3D-rendered image can be seen in (**C**). When the adjustment screw (blue) is turned, the coil former (red) is lowered or lifted - stabilized by a guide bar (green) - and therefore changing the position of the RF coil array (white) with respect to the sample. The coil interface containing the power splitter and T/R switches is positioned under the rat.

The size of a rat’s head is highly dependent on the age and gender of the rat. A one-size-fits-all housing was designed, onto which the rat can be positioned and prepared for the measurement. With the head placed inside the coil opening, the height of the RF coil can be adjusted to suit the size of the rat. The housing and the components for the adjustment mechanism were designed using 3D CAD software (Autodesk Inventor Professional 2021, Autodesk Ireland Operations Ltd., Dublin, Ireland) and 3D printed using selective laser sintering of biocompatible polyamide 1101 (Shapeways, New York, NY, USA), as shown in Figure 1. The coil former consists of an elliptically shaped top part, which is extended towards the bottom (Figure 1, red part). The ellipse has semi-major and semi-minor axes of 23 mm and 17.5 mm, respectively. The coil opening can be adjusted in height from 17.5 mm to 45 mm using an adjustment screw (Figure 1, blue part). The coil former movement is stabilized by a guide bar (Figure 1, green part).

**Figure 2:**
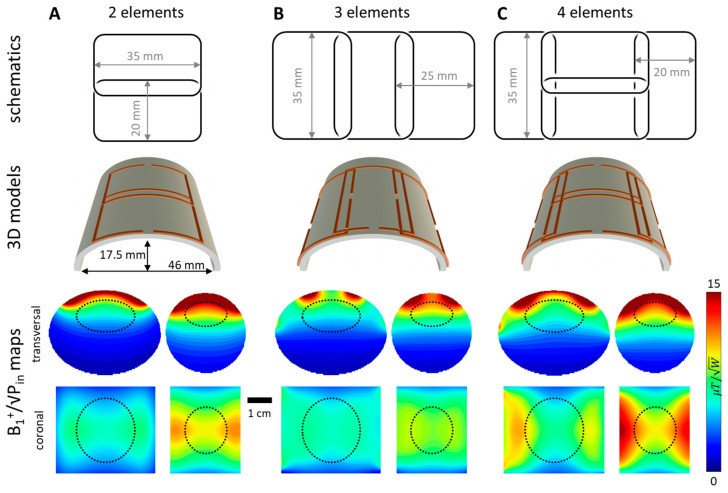
The three investigated array designs comprising 2, 3, and 4 elements in (**A**), (**B**) and (**C**), respectively. The corresponding ellipsoidal brain ROIs for the large phantom and the small phantom are indicated by a dotted black line.

The previously described simulation comparison resulted in a final 4-element array design comprising 35 × 20 mm^2^ elements arranged according to Figure 2C. Geometric overlap decoupling was chosen for all adjacent neighboring elements and was adjusted experimentally. Additionally, inductive decoupling was realized using counter-wound inductances to minimize mutual flux between the non-overlapping side elements.

### 2.3. Bench Tests

Quality factors were measured for the loaded (Q_l_) and unloaded (Q_u_) case with a double pickup coil [36]. The RF coil was constructed using 1 mm thick copper wire. Tuning was achieved using variable capacitors (TG092 0.5-10 pF, Exxelia Temex, Pessac, France) in parallel to the coil wire, and matching to 50 Ω was accomplished using fixed ceramic chip capacitors (CPX Series, Exxelia Temex, Pessac, France). Decoupling inductors were manually wound using copper wire with a diameter of 1 mm. To connect the coil to the scanner, an interface containing a four-way Wilkinson power divider and four transmit/receive (T/R) switches based on fast-switching PIN diodes (DH80106-10N, Cobham, Wimborne, UK) was built. The respective phase offsets for the static B_1_^+^ shim were implemented by coaxial cables of different lengths connecting the individual elements with the respective T/R switch. Scattering (S-) parameters were measured on a vector network analyzer (E5071C and E5092A; Agilent, Santa Clara, California, CA, USA). In order to prevent common mode currents, solenoidal cable traps tuned to the Larmor frequency were placed between each coil′s matching network and the respective T/R switch.

### 2.4. MR Experiments

An ex vivo measurement was performed on an adult female Sprague Dawley rat (aged 4 months, weighing 558 g). MR measurements were conducted on a 7 T whole-body MR scanner (7 T Magnetom, Siemens Healthineers, Erlangen, Germany) equipped with an SC72d gradient coil (maximum gradient strength 70 mT/m, slew rate 200 T/m/s). A 3D gradient echo image (TR/TE = 137/5.4 ms, resolution = 0.2 × 0.2 × 1 mm^3^, 5 averages, acquisition time = 1:54 min) as well as flip angle maps using a saturated turbo fast low angle shot (FLASH) sequence [37] (excitation pulse amplitude V_ref_ = 10 V, rectangular pulse shape, pulse duration = 500 μs, matrix size = 64 × 64, FOV = 50 × 50 mm^2^, 1.5 mm slice thickness) were acquired.

## 3. Results

### 3.1. EM Simulations

The schematics, 3D models, and simulated B_1_^+^ maps of the investigated array designs loaded by either a small cylindrical or a big elliptic cylindrical phantom roughly representing a juvenile and an adult rat head can be seen in Figure 2. The four-element array exhibits the highest mean B_1_^+^ and SAR efficiency in the brain ROIs (black dotted areas in Figure 2 for both the small and large phantom). Accordingly, the four-element design was chosen for practical implementation. Table 1 summarizes the resulting values for all investigated designs.

**Figure 3:**
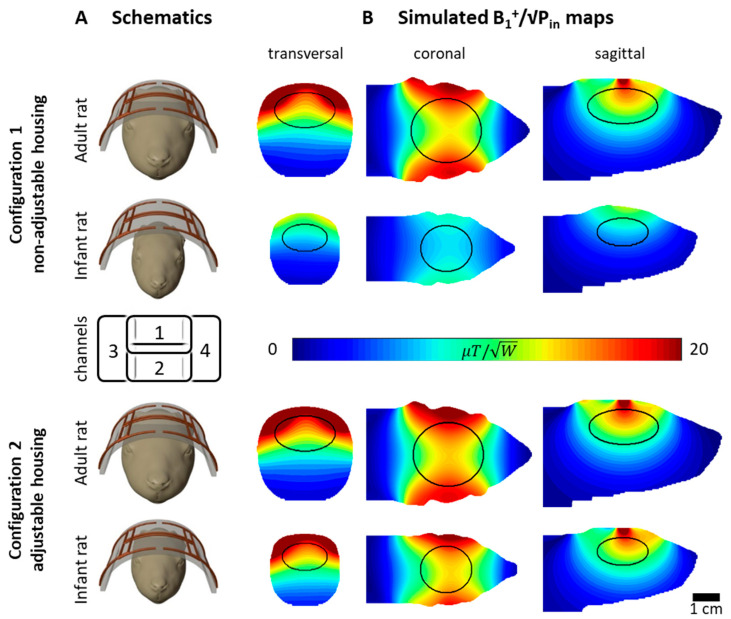
Simulated B_1_^+^ maps for adult and infant rats and loading conditions for configurations 1 and 2, representing non-adjustable and adjustable housing, respectively. A schematic representation can be seen in column (**A**). Static B_1_^+^ shim maximizing SAR efficiency was derived for each configuration and resulting B_1_^+^ maps can be seen in column (**B**). The corresponding phase offsets are stated in the text. Ellipsoidal brain ROIs used for coil performance evaluation are marked by black lines.

Figure 3 and Table 2 show the influence of different loading conditions. Configuration 1 represents a non-adjustable housing, resulting in an air gap between the coil and the sample when the RF coil is loaded with an infant rat. Configuration 2 represents the proposed adjustable housing, which brings the RF coil former closer to the infant rat.

In the simulation, tuning and matching were optimized for each configuration with the adult and the infant rat S-parameters set as combined optimization targets, naturally resulting in sub-optimal S-parameters for configuration 1, as seen in Table 2. Sub-optimal reflection coefficients (S_ii_ < −3.6 dB and S_ij_ < −10.0 dB) were found for configuration 1, while acceptable values for both sample sizes were obtained for configuration 2 (S_ii_ < −7.5 dB and S_ij_ < −12 dB); see Table 2.

The B_1_^+^ shimming phase set that maximizes SAR efficiency in the brain ROI was derived for each configuration and resulted in an optimal relative phase shift of channels 1, 2, 3, and 4 of (0°, 0°, 80°, −80°) and (0°, 0°, 65°, −95°) for configurations 1 and 2, respectively. The resulting B_1_^+^ maps can be seen in Figure 3B. With an input power of 1 W, the mean B_1_^+^ values in the brain ROI for configuration 1 were 11.7 µT/√W in the adult rat and 8.8 µT/√W in the infant rat. In configuration 2, the resulting power efficiency in the ROI was 13.7 µT/√W in the adult and 16.4 µT/√W in the infant rat. The mechanical adjustability of the housing therefore resulted in a B_1_^+^ increase of 17.3 % and 86.7 % for the adult and infant rats, respectively.

### 3.2. Bench Measurements

The average Q-factor for the individual elements in the unloaded case (Q_u_) was 395. The loaded Q_l_ was 76 for the two top elements (channels 1 and 2) and 122 for the two side elements (channels 3 and 4). The reason for the slightly higher Q in the side elements is that they are less form-fitted. This results in a Q-ratio (Q_u_/Q_l_) of 5.2 and 3.2, respectively, indicating sample noise dominance despite their small size in both cases. Matching below −20.2 dB and isolation between all array elements below −10.1 dB was achieved. The full measured S-parameter matrix can be seen in Table 3.

### 3.3. MR Measurements

**Figure 4:**
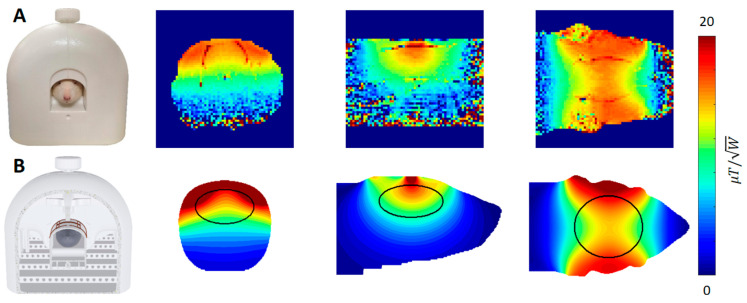
Measured (**A**) and simulated (**B**) B_1_^+^ maps. The top row shows the calculated B_1_^+^ maps derived from the flip angle measurements with an ex vivo rat. The bottom row shows the simulated results for the adult rat. The maps are quantitatively and qualitatively highly comparable.

The simulated and measured B_1_^+^ maps derived from the flip angle measurements for an ex vivo adult rat can be seen in Figure 4. The simulation is highly comparable, both quantitatively and qualitatively, to the acquired B_1_^+^ maps. Discrepancies in the maximum B_1_^+^ are in the order of 15 %, which can be attributed to the differences between the used voxel model and the ex vivo rat.

**Figure 5:**
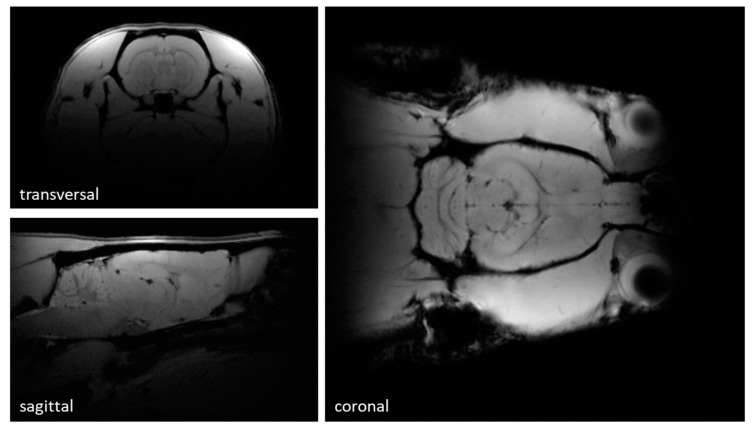
Gradient echo images of an ex vivo Sprague Dawley rat in transversal, sagittal, and coronal direction, showing good brain coverage. In-plane resolution is 0.2 × 0.2 mm^2^ with a slice thickness of 1 mm. Prominent structures like the cerebellum and cerebrum are homogenously covered by the array’s excitation and reception field.

Slices in transversal, sagittal, and coronal orientation through the rat’s brain from 3D gradient echo data can be seen in Figure 5. The images acquired have a resolution of 0.2 × 0.2 × 1 mm^3^, and enable a clear depiction of the rat brain anatomy.

## 4. Discussion

In this work, we present a dedicated four-channel transceiver coil array, specifically developed for rat brain MRI with a human whole-body 7 T scanner. The housing was designed and 3D-printed to be mechanically adjustable in order to facilitate the scanning of rats of different ages and, thus, head sizes and shapes. This adjustability ensures similar matching conditions despite different head sizes. The element size and wire width were chosen so that the elements would still be dominated by sample noise, indicating that cooling of the coil would not provide strong SNR gain.

Although the relative phase offsets for B_1_^+^ shimming are fixed by the cable lengths from the interface to the coil elements, the chosen phase set for the four-channel configuration achieves good simulation results in both the small and big rats. When comparing the two- to four-channel array designs, the power and SAR efficiency are always an ascending function of the number of elements, except for the three-channel configuration’s power efficiency simulated for the small phantom. This could be explained by a potentially higher coupling between elements and lower coil loading in this case.

In our study, the static B_1_^+^ shim and coil array element arrangement is optimized with regard to the maximum achievable B_1_^+^ and SAR efficiency and not homogeneity. Even if the qualitative analysis of the B_1_^+^ field distribution shows less homogeneity for the four-channel array than the two-channel array, it is considered homogeneous enough for the target application of rat brain imaging. Given ample signal intensity using the four-channel array (see Figure 5), inhomogeneities could be remedied with appropriate post-processing techniques, e.g., signal intensity correction [38].

The mechanical adjustment mechanism for the vertical positioning of the four-element array enables the RF coil to be placed directly above the rat’s head, increasing the simulated mean B_1_^+^ in the brain ROI for the infant rat by +86.7 % and for the adult rat by +17.3%. Arguably, the position change could also be achieved by positioning smaller rats on a specifically designed spacer in order to bring them closer to the array. However, this method tends to be less stable, and positioning of the required nose cone for anesthesia might be difficult or even impossible. Also, dedicated spacers for various rat sizes would be required. In addition, the possibility of enlarging the coil opening is useful for easy positioning of the rat within the coil, while the individual adjustment of the height can be achieved when the rat and the nose cone are correctly positioned. Our proposed adjustment mechanism enables continuous vertical adjustment and maintains space for the nose cone at all times.

A limitation of this work is the non-availability of infant rats on-site, meaning that there are no bench or MR measurements, which would have enabled the performance with different loading conditions to be thoroughly demonstrated. As the coil tuning, matching, and decoupling (S_ii_ and S_ij_) were jointly optimized for the adult and infant rat in the co-simulation algorithm but adjusted to the adult rat only on the bench, the simulated and measured S-parameter results show a strong deviation. Ideally, in practice, the S_ii_-parameters would remain similar for the infant and adult real rat measurement with our adjustable coil design and comparable coil loading conditions. Potentially, a certain trade-off in matching levels would still have to be found, as implemented in the simulation optimization.

Measured and simulated B_1_^+^ maps are in good agreement. There is, however, a difference in the maximum B_1_^+^, close to the coil. This deviation is most likely mainly caused by the discrepancy between the simplified rat model and coil former used in the simulation and the anatomy of the real rat and final implemented housing design. Additionally, the fur introduces a distance between the rat and the coil which is not present in the simulation.

MR measurements in an adult rat show a highly optimized field of view of the rat’s brain, covering the brain but not extending too far below or besides, enabling efficient image acquisition.

The proposed coil design could allow sites without dedicated preclinical MRI scanners to conduct rodent studies on a human 7 T scanner. The four-channel coil is optimized in terms of transmit and receive performance with regard to safety constraints (minimum SAR) for rat brain MRI, but in principle, the coil could be used and further optimized, e.g., for whole-body mouse imaging.

In conclusion, a dedicated four-channel 7 T transceiver RF coil array that is suitable for performing preclinical studies on the brains of rats of all sizes was designed, built, and evaluated in simulations and bench and MR measurements.

## Figures and Tables

**Table 1:** Power and SAR efficiency for the 2-, 3- and 4-element array loaded by cylindrical and elliptical phantom. The mean B_1_^+^ was evaluated in the corresponding brain ROI (see Figure 2). For SAR efficiency calculations, the maximum 1 g averaged SAR over the whole phantom was used.

	Small Phantom			Large Phantom		
	2	3	4	2	3	4
	Elements			Elements		
**Power efficiency** **[μT/√W]**	10.1	9.1	10.2	5.8	6.1	6.7
**SAR efficiency** **[μT/√(W/kg)]**	0.9	1.1	1.3	0.6	0.9	1.0

**Table 2:** Simulated S-parameters (in dB) corresponding to the configurations shown in Figure 3. Channel (ch) indices correspond to the array channels shown in Figure 3.

	Simulated S-Parameters [dB]									
	Adult Rat					Infant Rat				
**Configuration 1** **non-adjustable housing**	**ch**	**1**	**2**	**3**	**4**	**ch**	**1**	**2**	**3**	**4**
	1	−10.5				1	−5.3			
	2	−20.4	−4.7			2	−10.0	−3.6		
	3	−26.0	−14.4	−11.8		3	−33.8	−36.0	−11.7	
	4	−26.2	−14.6	−11.9	−11.9	4	−33.6	−35.8	−12.5	−11.9
**Configuration 2** **adjustable** **housing**	**ch**	**1**	**2**	**3**	**4**	**ch**	**1**	**2**	**3**	**4**
	1	−11.4				1	−10.8			
	2	−19.4	−10.5			2	−16.6	−7.5		
	3	−25.7	−14.4	−11.9		3	−30.2	−19.4	−11.8	
	4	−25.7	−14.8	−19.7	−11.9	4	−30.5	−19.5	−12.0	−11.8

**Table 3:** Measured S-parameter matrix of the RF coil array loaded with an adult rat.

Measured S-Parameters [dB]				
ch	1	2	3	4
1	−23.0			
2	−18.5	−26.3		
3	−14.7	−12.6	−20.2	
4	−15.8	−10.1	−14.7	−27.3

## Data Availability

The data presented in this study are available on request from the corresponding author. The data are not publicly available due to legal restrictions.
